# Microcirculatory changes in the skin after postmastectomy radiotherapy in women with breast cancer

**DOI:** 10.1038/s41598-024-54650-4

**Published:** 2024-02-20

**Authors:** Sherif Elawa, Robin Mirdell, Aristotelis Stefanis, Erik Tesselaar, Simon Farnebo

**Affiliations:** 1 Department of Biomedical and Clinical Sciences, Faculty of Medicine and Health Sciences, Linköping University, 58185 Linköping, Sweden; 2 Department of Medical Radiation Physics, Department of Health, Medicine and Caring Sciences, Linköping University, Linköping, Sweden; 3 Department of Plastic Surgery, Hand Surgery, and Burns, Linköping University, Linköping, Sweden; 4 Department of Clinical Chemistry, Linköping University, Linköping, Sweden

**Keywords:** Breast cancer, Optical techniques

## Abstract

Postmastectomy radiotherapy (PMRT) increases the risk for complications after breast reconstruction. The pathophysiological mechanism underlying this increased risk is not completely understood. The aim of this study was to examine if there is a relationship between PMRT and microvascular perfusion in the skin directly after, and at 2 and 6 months after PMRT and to assess if there is impaired responsiveness to a topically applied vasodilator (Methyl nicotinate—MN) after PMRT. Skin microvascular responses after PMRT were measured on two sites in the irradiated chest wall of 22 women before, immediately after, and at 2 and 6 months after unilateral PMRT with the contralateral breast as a control. A significant increase in basal skin perfusion was observed in the irradiated chest wall immediately after RT (*p* < 0.0001). At 2 and 6 months after RT, there was no longer a difference in basal skin perfusion compared to the contralateral breast and compared to baseline. Similarly, the blood flow response in the skin after application of MN was stronger immediately after RT compared to before RT (*p* < 0.0001), but there was no difference at later time points. These results indicate that the increased risk for complications after surgical procedures are not directly related to changes in skin perfusion and microvascular responsiveness observed after postmastectomy RT.

## Introduction

Postmastectomy radiotherapy (PMRT) has been shown to decrease local recurrence and improve survival in women with node-positive breast cancer^1^. It reduces the risk of local recurrence and improves overall survival by 24% in woman^2^.

Besides preventing recurrence of breast cancer, PMRT usually causes side effects in the skin. Up to 90% of patients experience acute dose-dependent skin reactions in treated areas, ranging from mild erythema to severe ulcerations^1^. Late possible chronic injury includes skin atrophy, dryness, telangiectasia, dyspigmentation, dyschromia, tissue scaring and fibrosis^3–5^. The chronic changes that lead to radiation induced fibrosis (RIF) can take months to years before full manifestation^6^.

It has been speculated that the radiosensitivity of the skin is due to its high proliferative capacity and oxygenation requirements of its basal epidermal cells^7^, potentially secondary to hypovascularity and development of chronic tissue hypoperfusion. An impaired microcirculatory function is further thought to be a potential cause for wound infections and implant extrusion in patients that is breast reconstructed and previously received PMRT. The exact mechanism of radiation-induced injury is however not completely understood. On a molecular level, ionizing radiation (IR) induces several types of cell damage^8^. The resulting cellular death activates the immune system with an additional antitumor response^9,10^. Ionizing radiation generates reactive oxygen species (ROS) in the nucleus which also contributes to cellular damage and death by apoptosis^11,12^.

Many patients who have undergone mastectomy with subsequent radiotherapy later desire restoration of the breast. Successful breast reconstruction strongly relies on a functional vascular bed in the reconstructed tissue, both when implants are used and with autologous breast reconstruction. The skin microcirculation has been regarded as a representative vascular bed for the assessment of tissue microvascular function^13^. Measurable effects on skin microcirculation that are associated with PMRT could be a marker for impaired healing properties, and may indicate an increased risk for ensuing tissue morbidity when reconstruction of the breast is done. Assessing the effects of radiation on the skin microcirculation after resection may thus help decide which patients are at risk for complications after specific reconstruction procedures.

The aim of this study was therefore to (1) examine if there is a relationship between PMRT and microvascular perfusion in the skin directly after, and at 2 and 6 months after PMRT and to (2) assess if there is impaired responsiveness to a topically applied vasodilator (Methyl nicotinate—MN) after PMRT, and also to (3) explore whether microcirculatory changes after PMRT are dependent on co-morbidity factors including; radiation dose, age, hypertension or previous adjuvant chemotherapy.

## Method

### Participants

Between February 2019 and June 2020, we enrolled 22 women, mean (SD) age 62.4 years (9.3 years), with breast cancer who had undergone macroscopically complete surgical removal of the tumour and required PMRT. Five women (23%) received a conventional regimen of 25 × 2 Gy, while 17 (77%), underwent a hypofractionated regimen of 16 × 2.66 Gy. Additionally, boost radiation was administered in 2 (9%) of the cases. Various energy levels were used, with 6 MV being the most common in 9 (41%) patients, followed by a combination of 6 + 15 MV in 8 (36%) patients, and 15 MV alone in 5 (23%) patients.

The number of participants was based on a sample size that allows for detecting a 20% change in microvascular perfusion, accounting for a 10% loss to follow up. Before inclusion, the participants gave written informed consent. All were asked to abstain from caffeine for at least 24 h before the measurement. Measurements were made at a room temperature of 21.0 ± 1.0° C with the participants in a supine position. The study was carried out according to the Declaration of Helsinki and was approved by the Regional Ethics Committee at Linköping University Hospital, Dnr 2014/299-31.

### Equipment

A Laser Speckle Contrast Imager (PeriCam PSI System, Perimed AB, Järfälla, Sweden) was used to measure the perfusion of the skin. The measurement principle of LSCI has previously been described in detail^13^. This system uses a divergent class 1 laser with a wavelength of 785 nm that illuminates the skin. The laser light creates a fluctuating speckle pattern due to optical interference. Due to the finite exposure time of the camera, these fluctuations result in a decrease in the contrast between the speckles. The local speckle contrast in the image is therefore correlated to the perfusion in the skin. From this pattern, a perfusion value is calculated, given as perfusion units (PU), an arbitrary unit proportional to the concentration and mean velocity of red blood cells. The same acquisition parameters were used for all perfusion measurements. The measurement distance was between 20 and 30 cm. The sampling rate was set to 21 images per second, 5 images were used to construct an average image giving an effective frame rate of 4.2 images per second. The system was calibrated at regular intervals as recommended by the manufacturer.

### Experimental procedure

**Figure 1:**
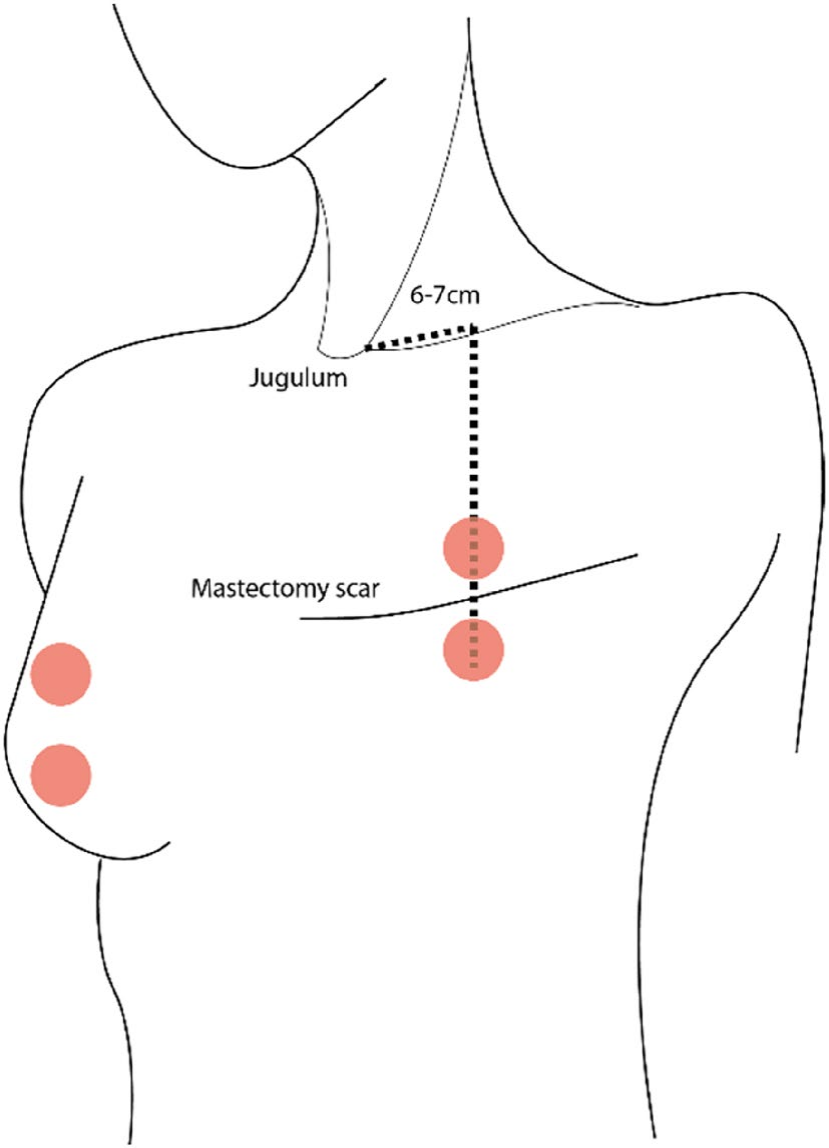
Illustration of the measurement sites. Skin perfusion before and after application of methyl nicotinate was measured on the midbreast, both above and below the areolar complex or mastectomy scar.

Chlorhexidine ethanol (5 mg/ml, Fresenius AB, Uppsala, Sweden) was used to clean the skin before the experiments. Thereafter, 50 μl 20 mM of methyl nicotinate (MN, Sigma-Aldrich, Merck Group) was applied on circular a 3-cm area of the skin above and under the areolar regions of both chest wall and contralateral breast, using a pipette. Then, the MN was gently rubbed into the skin by hand while wearing latex gloves. Baseline perfusion was measured for one minute on each site before applying the drugs. After 15 min of MN application, perfusion was again measured for one minute (Fig. 1).

The measurements were done immediately before the start of radiotherapy, directly after the last fraction of radiotherapy, and at 2 months and 6 months after radiotherapy.

### Data analysis

Skin perfusion images were analysed using the LSCI system’s software (PimSoft 1.5, Perimed AB, Järfalla, Sweden). Regions of interest (ROI) were selected manually in the first image of each series, perfusion at rest without MN, with one ROI above and one below the areola and on the contralateral breast. When the areola was missing, ROI were placed midclavicularly above and below the mastectomy scar. Then, the locations of ROI in subsequent images (after application of MN) were verified and corrections for movement of the patient were made as needed. A two-way ANOVA for repeated measures was used to evaluate the difference in perfusion across different time points, between different radiation doses and between different measurement sites.

As there were no significant differences in perfusion between ROIs above and below the areola, the mean of the two ROIs was used in the analysis of perfusion and of the perfusion response after application of MN.

Multiple linear regression was used to explore the influence of potential factors associated with the microvascular response after RT (Radiotherapy). Baseline perfusion and perfusion response after MN application in the irradiated chest wall at the different time points were used as the outcome variables. Age, BMI, blood systolic and diastolic pressure, adjuvant medical treatment, hypertension, smoking, diabetes mellitus, and radiation dose were assumed factors that could be of influence (independent variables). The backward stepwise selection method was used to produce an initial screening of these potential factors. At each step, the *p* value of an *F*-statistic was used to check whether the model was statistically significant. A cut-off value of > 0.1 was used as exclusion criterium.

Matlab (R2019a, The MathWorks, Natick, MA, USA) was used for the multiple regression. Other statistical calculations were made using GraphPad Prism (version 6 for Windows, GraphPad Software, San Diego, CA, USA), and a probability of less than 0.05 was accepted as significant. For the stepwise regression method, p-values are generally considered invalid, and were therefore not reported.

## Results

Descriptive data on the study participants is shown in Table 1. Mean perfusion at all timepoints is shown in Table 2.

**Table 1:** Descriptive data on the study participants (n = 22). Data are presented as mean SD or n (%).

Number of women included	22
Radiation therapy details	n (%)
25 × 2 Gy	5 (23)
16 × 2.66 Gy	17 (77)
Boost	2 (9)
Bolus	1 (5)
6 MV	2 (9)
6 + 15 MV	19 (86)
15 MV	1 (5)
Physiological parameters	mean (SD)
Age (years)	62.4 (9.3)
Length (cm)	164.7 (5.2)
Weight (kg)	75.8 (15.8)
Systolic blood pressure (mmHg)	130 (15)
Diastolic blood pressure (mmHg)	79 (9.8)
Comorbidities	n (%)
Smoking	3 (13)
Diabetes Mellitus Type 2	2 (9)
Hypertension	6 (26)
Inflammatory bowel disease	1 (4)
Cardiovascular disease	0 (0)
Medication	n (%)
Warfarin	1 (4)
ASA	2 (9)
Adjuvant chemotherapy	11 (48)
Letrozole	16 (70)
Tamoxifen	4 (17)

**Table 2:** Mean perfusion at rest (upper) and after application of methyl nicotinate (lower) in irradiated and non-irradiated skin before start of radiotherapy and at 3 time points after radiotherapy.

	Mean Perfusion at rest (PU)					
	Irradiated			Non-irradiated		
	All	42.56 Gy	50 Gy	All	42.56 Gy	50 Gy
Before RT	76 (13)	75 (11)	81 (22)	76 (11)	76 (11)	74 (18)
Directly after RT	119 (45)	110 (41)	178 (43)	81 (26)	83 (28)	92 (26)
2 months	85 (17)	84 (17)	97 (28)	77 (13)	75 (12)	84 (16)
6 months	84 (15)	85 (14)	83 (30)	77 (13)	80 (12)	63 (6)

	Mean Perfusion after methl nicotinate (PU)					
	Irradiated			Non-irradiated		
	All	42.56 Gy	50 Gy	All	42.56 Gy	50 Gy
Before RT	162 (53)	162 (52)	149 (66)	160 (47)	158 (48)	165 (47)
Directly after RT	184 (38)	183 (36)	185 (54)	162 (42)	160 (44)	171 (38)
2 months	169 (45)	166 (48)	172 (40)	161 (43)	157 (45)	177 (31)
6 months	163 (45)	159 (44)	189 (58)	159 (45)	156 (45)	175 (50)

**Figure 2:**
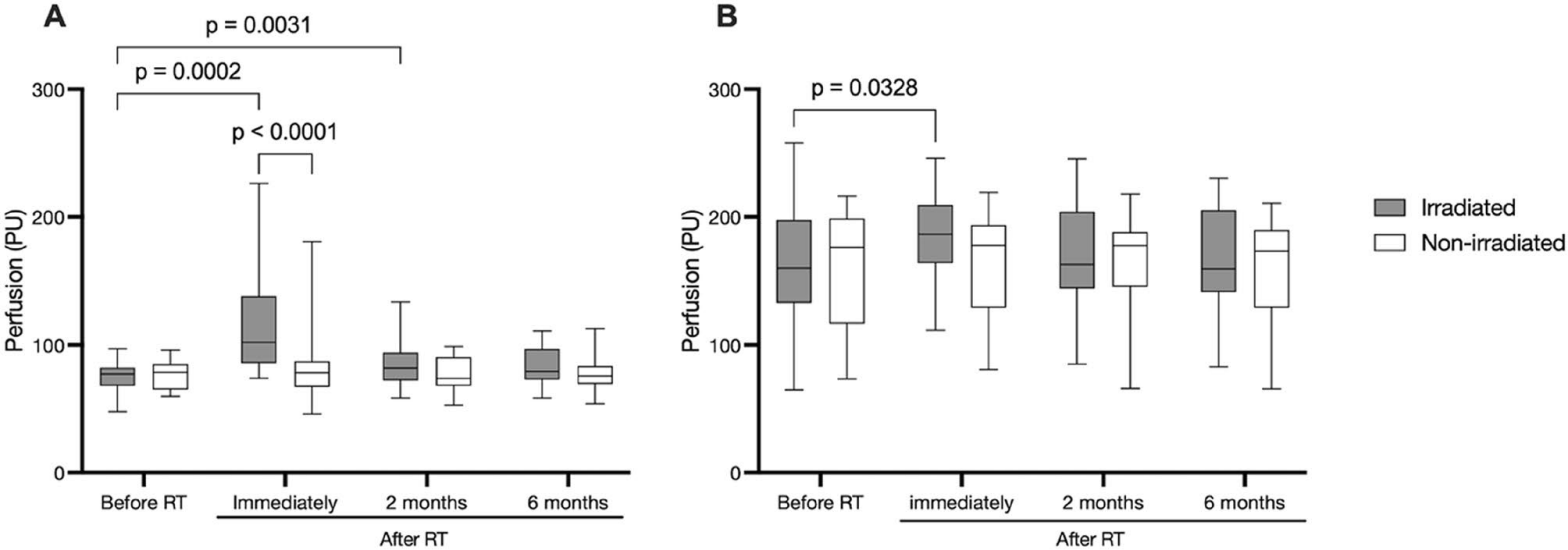
**(A)** Mean perfusion at rest in irradiated and non-irradiated skin before RT and at 3 time points thereafter. **(B)** Mean perfusion in irradiated and non-irradiated skin after application of MN before RT and at 3 time points thereafter.

**Figure 3:**
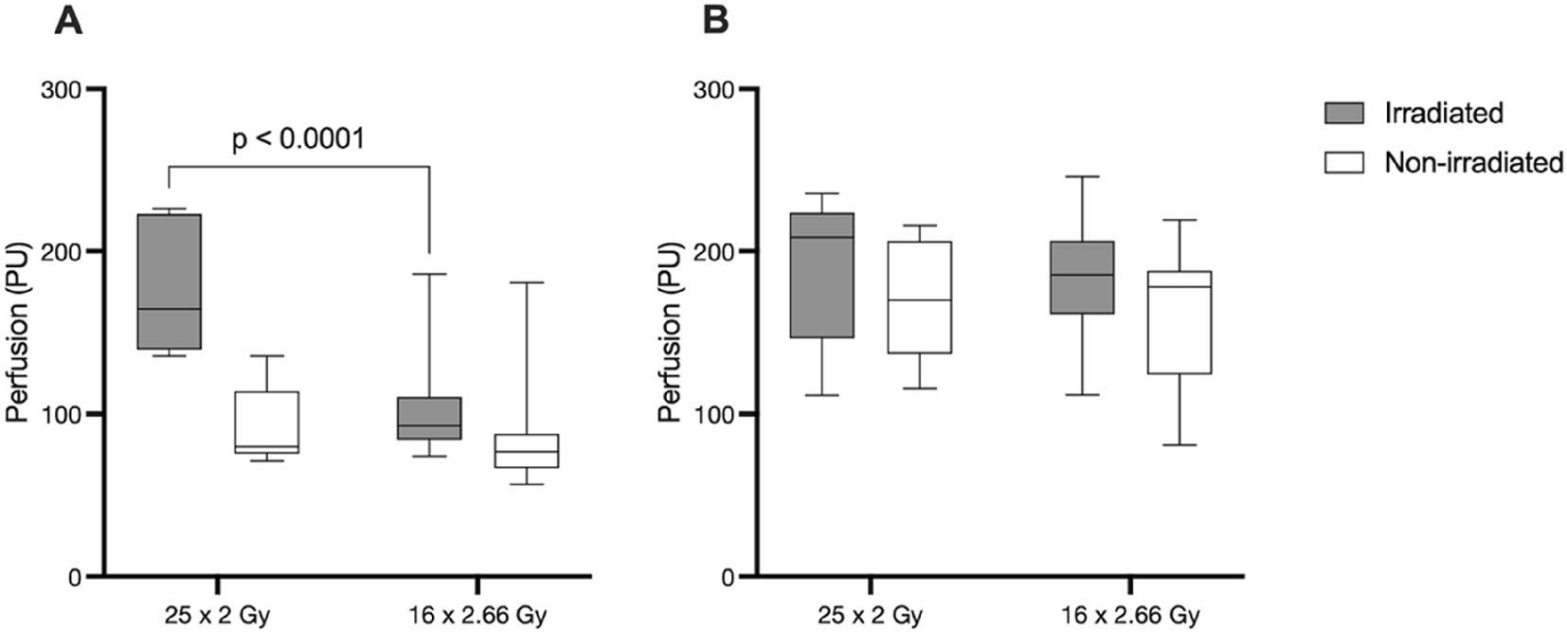
**(A)** Mean perfusion at rest in irradiated chest wall and non-irradiated breast after the last fraction of PMRT, in women receiving prolonged radiation protocol of 50 Gy (25 × 2 Gy), including patients receiving boost, and short radiation protocol of 42.56 Gy (16 × 2.66 Gy). **(B)** Mean perfusion in irradiated chest wall and non-irradiated breasts after the last fraction of PMRT, in women receiving a prolonged radiation protocol of 50 Gy (25 × 2 Gy), including patients receiving boost, and short radiation protocol of 42.56 Gy (16 × 2.66 Gy) after topical application of MN.

There was no significant difference in skin perfusion between measurement sites above or below the areolar complex/mastectomy scar, nor was there a difference in skin perfusion between the irradiated chest wall and non-irradiated breast before RT, at 2 months after RT, or at 6 months after RT. Immediately after RT, a significant increase in skin perfusion was noted in the irradiated chest wall (119 (45) vs. 76 (13) PU, *p* < 0.0001) whereas the perfusion in the non-irradiated breast was unchanged (81 (26) vs. 76 (11) PU). At two months after RT, there was still a small but significant increase in perfusion compared to before RT (85 (17) vs. 76 (11) PU, *p* = 0.0031) (Fig. 2a). In women who received a prolonged radiation protocol, a significantly larger increase in perfusion was observed directly after RT in the irradiated chest wall (25 × 2 Gy: 178 (73) PU vs. 16 × 2.66 Gy: 110 (41) PU *p* < 0.0001) but no differences were observed at other time points (Fig. 3a).

After MN was applied, a significant increase in skin perfusion was measured at all time points, both in the irradiated chest wall and non-irradiated breast. In the irradiated chest wall, the perfusion after application of MN was significantly higher immediately after RT compared to before RT (*p* = 0.0328) (Fig. 2b). No effect of radiation dose on the perfusion response to application of MN could be observed (Fig. 3b).

The backwards stepwise logistic regression model identified three factors that influenced the microvascular response. Across all measurement points, diabetes mellitus was identified as a factor resulting in reduced vasodilatory response after radiation, whereas hypertension and a higher radiation dose were factors that were related to increased vasodilatory response.

## Discussion

The main finding in this study is that the marked increase in skin perfusion in the irradiated chest wall immediately after PMRT is reduced to baseline levels two months after PMRT treatment. After six months, the skin perfusion at rest, and after provocation with MN showed the same results as before PMRT and 2 months after PMRT. The microvascular reactions in the skin to PMRT are therefore reversible earlier than previously described^1,3–7^. These findings suggest that the effects of PMRT on impaired healing properties and risk for ensuing tissue morbidity with subsequent surgical procedures cannot simply be explained by changes in skin microcirculation.

We have used LSCI as an objective non-invasive tool to follow microcirculatory changes due to PMRT. LSCI is based on the speckle pattern created on a surface by a laser source that contains information about the speed and concentration of erythrocytes in the superficial capillaries of the skin. The pattern is analyzed by a computer that presents an arbitrary value of the perfusion (PU). This technique has gained significant interest due to its applicability in imaging perfusion and predicting tissue morbidity in; burn wounds, retinas, brain, liver, oesophagus, and large intestines^14–20^. For reconstructive surgeons, LSCI can be a valuable tool in clinical decision-making both per-operatively and postoperatively^16,17,21^. LSCI has been shown to provide reliable data on tissue perfusion in compromised parts of flaps due to arterial and venous occlusion as well as predicting tissue necrosis with high accuracy^16,17^. It has also been shown to correlate well with other well-established techniques, such as laser doppler flowmetry (LDF)^16^ and indocyanine green fluorescence angiography (ICG-FA)^22^. It is likely that the technique may also be valuable in pre-operative assessment, to determine if there are limitations in microcirculatory capacity that may endanger most planned reconstructive flap procedures involving exposed skin.

One approach to improve assessment of microvascular function in tissues in the resting state is by increasing its basal perfusion so that its maximum capillary capacity is reached. The use of topical application of MN to increase basal perfusion in the skin has been evaluated with LSCI previously^14,15^. It has been proposed that the perfusion dynamics seen during such provocations correspond to its ability to withstand stress at time of surgery, however this has not been clinically proven.

Tesselaar et al. studied the acute effects in skin microcirculation during radiation therapy for breast cancer with laser Doppler flowmetry, laser speckle contrast imaging, and polarized light spectroscopy imaging. Instead of MN, which initiates a prostaglandin mediated vasodilation, they used local heating to measure endothelium-dependent vasodilation^23^. Skin reactions, with and without MN, in our study population immediately after PMRT showed the same result as in that paper. There were significant increases in blood flow and velocity in the superficial vascular beds on the irradiated side, both at baseline and after stimulating a vasodilatory response with MN. These immediate effects in skin microcirculation can be explained by a radiation induced series of events, including signalling between cells in the epidermis and dermis, and a cascade of cytokines and chemokines^8,12^. The measured skin reactions on the irradiated side immediately after PMRT were significantly higher, both with and without MN provocation, than the measurement two and six months after the last PMRT session.

MN is a safe and easy-to-use method for the evaluation of the reactivity of the microcirculation in the skin, as it provides a reliable and strong hyperaemic plateau phase 15 min after application^15^. Still, according to our data, the microvascular hyperaemic response to MN does not seem to be completely saturated. We noted that the increase in blood flow and velocity in the superficial vascular bed on the irradiated side remained dose dependent after stimulation with MN. Therefore, MN seems to be a reliable method to measure a hypothetical irradiation induced damage to the superficial vascular bed. Two and six months after irradiation there is no proof, with or without MN, that total capillary capacity is affected on the irradiated side compared to the non-irradiated side. Total capillary capacity does therefore not seem to be a good measurement for patient morbidity after PMRT.

Our multivariate analysis indicated that the vasodilatory response is positively associated with the delivered radiation dose. This is in conjunction with the observation that the degree of radiation injury is related to the total radiation dose and the time interval of received radiation dose^24^. Furthermore, hypertension was identified as a factor that increases the vasodilatory response after RT. This is interesting as studies on skin PORH (post occlusive reactive hyperaemia) with LSCI displayed a significantly impaired microvascular function in patients with essential hypertension and patients with masked hypertension compared to those in women without hypertension^25^. However, whether blood pressure-lowering therapy improves skin microvascular function in patients with hypertension has not been investigated in previous studies. Patients in our study with hypertension were under pharmacological treatment with well-adjusted blood pressure.

Although BMI has previously been found to be associated with increased risk of acute skin toxicities during radiation treatment for early-stage breast cancer^21^, it was not identified as a predicting factor in the current study. This may be caused by the limited sample size.

The reduced vasodilatory response to PMRT in our subgroup of patients with diabetes mellitus can be due to increased inactivation of nitric oxide or to decreased reactivity of the vascular smooth muscle to nitric oxide as described by Williams et al.^26^.

This study highlights that there is a remaining significant knowledge gap regarding the impact of PMRT on chest wall tissue, as we did not observe any effects on microvascular function six months after PMRT. Clinical evidence suggests that breast implant reconstruction in patients receiving PMRT is associated with high rates of acute and chronic complications, as well as poor aesthetic outcomes^27^. Vascular effects and tissue fibrosis are considered potential causes for morbidity in these patients. One proposed model suggests that tissue fibrosis leads to tissue hypovascularity, resulting in decreased delivery of oxygen and nutrients. This, combined with radiation-induced vascular lesions, is hypothesized to be the main cause of tissue radiation injury^6,28–31^. The role of tissue fibrosis on soft tissue microvasculature following radiation was examined by Rodriguez and colleagues in a murine model, who demonstrated early effects in the radiated skin including decreased vessel separation and increased vessel number and volume compared to non-radiated skin. Late effects showed a trend of decreased skin vascularity after radiation, with increased vessel separation and decreased vessel number compared to the control group^27^. Chin et al., using hyperspectral imaging, showed a decline in skin perfusion in irradiated mice, along with a reduction in blood vessel density and expression of vascular endothelial growth factor and its receptor^28^.

From a microcirculatory perspective, our study did not demonstrate any significant lasting effects on skin perfusion measured non-invasively after PMRT. However, the high complication rates after PMRT may be primarily attributed to tissue fibrosis. Radiation-induced fibrosis (RIF) involves progressive deposition of extracellular matrix (ECM) in interstitial and microvascular spaces^29^. The relationship between fibrosis and tissue hypoxia is debated, as oxygen is crucial for adequate radiation response, and hypoxia is postulated to be a major source of radiation resistance^30^. Mechanistically, radiation skin injury is a complex process involving an imbalance of antioxidant status, redox control of wound healing, and chronic inflammation. Radiation-induced skin fibrosis results from chronic inflammation and immune imbalance, leading to the release of profibrotic cytokines^6,31^. Therefore, measuring tissue oxygenation may be a more accurate way to identify radiation-induced fibrosis that could potentially lead to complications in subsequent reconstructive surgery.

It is important to note that this study has limitations. Microvascular changes were only assessed up to 6 months after postmastectomy radiotherapy (PMRT), and was restricted to assessment of the skin alone. Effects on subcutaneous tissues cannot be assessed using this technique. Also, longer-term follow-up would have allowed for the evaluation of delayed effects on skin perfusion and microvascular responsiveness. The study solely relied on laser speckle contrast imaging (LSCI) as the measurement tool for assessing microvascular perfusion. While LSCI is a valuable technique, the use of additional complementary methods could have provided a more comprehensive understanding of microvascular changes. Finally, the stepwise regression method used for predictor selection may not guarantee the identification of the best predictors, and the relatively small sample size in our study may also impose limitations when investigating potential co-factors.

To conclude, our findings suggest that the microvascular changes caused by radiotherapy of the breast are reversed after six months. The increased risk for complications during subsequent surgical procedures in women who have received radiation therapy cannot be explained by early changes in microvascular blood flow or microvascular responsiveness. Further studies are needed to identify possible other mechanisms underlying the impaired tissue viability, including reduced tissue oxygenation or tissue fibrosis.

### Supplementary Information

Supplementary Information 1.

Supplementary Information 2.

## Data Availability

All data generated or analysed during this study are included in this published article and/or the supplementary file.
